# Step-down from high dose fixed combination therapy in asthma patients: a randomized controlled trial

**DOI:** 10.1186/1465-9921-13-54

**Published:** 2012-06-25

**Authors:** Alberto Papi, Gabriele Nicolini, Nunzio Crimi, Leonardo Fabbri, Dario Olivieri, Andrea Rossi, Pierluigi Paggiaro

**Affiliations:** 1 Research Center on Asthma and COPD, University of Ferrara, Via Savonarola 9, 44100, Ferrara, Italy; 2 Medical Affairs Department, Chiesi Farmaceutici, Parma, Italy; 3 Department of Internal and Specialistic Medicine, Section of Respiratory Diseases, University of Catania, Catania, Italy; 4 Section of Respiratory Diseases, University of Modena, Modena, Italy; 5 Department of Clinical Sciences, University of Parma, Parma, Italy; 6 Pulmonary Unit, University/General Hospital of Verona, Verona, Italy; 7 Cardio-Thoracic and Vascular Department, University of Pisa, Pisa, Italy

**Keywords:** Beclomethasone, Extrafine, Fluticasone, Formoterol, Salmeterol

## Abstract

**Background:**

Asthma guidelines suggest that therapy can be reduced once asthma is controlled. Despite these recommendations, asthmatic patients are seldom stepped down in clinical practice, and questions remain about when and how to reduce asthma therapy. The purpose of the present study was to evaluate lung function and asthma control in patients who were stepped down from the highest recommended dose of inhaled corticosteroid/long acting β_2_ agonist combination therapy.

**Methods:**

This was a prospective, randomised, controlled, two-arm parallel group study. Asthmatic patients who were fully controlled with a high daily dose (1000/100 μg) of fluticasone/salmeterol were randomly assigned to 6 months of open-label treatment with either 500/100 μg fluticasone/salmeterol Diskus daily or 400/24 μg extrafine beclomethasone/formoterol pMDI daily. The primary outcome was the change in morning peak expiratory flow (PEF) values between baseline and the end of treatment. The secondary outcomes included asthma control and exacerbation frequency.

**Results:**

Four hundred twenty-two patients were included in the analysis. The PEF values remained above 95% of the predicted values throughout the study. The end-study morning PEF rates showed equivalence between the groups (difference between means, 2.49 L/min; 95% CI, -13.43 to 18.42). No changes from baseline were detected in PEF and forced expiratory volume in 1 second measured at the clinics, in the symptom scores or in the use of rescue medication. Asthma control was maintained in 95.2% of the patients at 6 months. No significant differences between the groups were detected in any other parameter, including exacerbation frequency and adverse events.

**Conclusions:**

Stepping down patients whose asthma is controlled with the highest recommended dose of fluticasone/salmeterol to either 500/100 μg fluticasone/salmeterol daily or 400/24 μg extra-fine beclomethasone/formoterol daily provides comparable maintenance of lung function and asthma control.

**Trial registration:**

clinicaltrials.gov NCT00497237

## Background

International guidelines recommend a stepwise approach for managing asthma in adults and children over 5 years of age [1]. The Global Initiative for Asthma (GINA) guidelines [1] indicate that “asthma control” is the objective of treatment, i.e., achieving and maintaining the control of symptoms and normal activity levels, maintaining pulmonary function as close to normal as possible, preventing asthma exacerbations and asthma mortality and avoiding adverse effects from asthma medications. Maintenance treatment should be stepped up or stepped down to the minimum amount of medication necessary to maintain asthma control and minimise the potential for long-term side effects [1].

Inhaled corticosteroids (ICS) represent the cornerstone of maintenance treatment. If asthma control is inadequate with low-dose ICS, the addition of a long-acting β2-agonist (LABA) provides enhanced clinical benefits compared to increasing the ICS dosage [2-4]. ICS and LABAs have complementary clinical and pharmacological activities when administered together [5].

Although asthma guidelines advocate reducing treatment once asthma is well controlled [1,6], clinicians appear to be reluctant to reduce treatment. Both treatment prescription surveys and clinical trials indicate that a large proportion of asthmatic patients are over-treated and seldom stepped-down in clinical practice [7,8].

Relatively few studies have evaluated the best strategy for dose reduction [7,9-11]. Some studies have documented a better maintenance of asthma control when the patients who are treated with ICS/LABA combination treatments were stepped down to a lower dose of the ICS/LABA combination, as opposed to suspending the LABA while maintaining the ICS at the same dose [7,10,11]. This step-down strategy has the advantage of maintaining asthma control and lowering the dose of ICS [7]. Conversely, 2 studies have shown that stepping-down from a low dose of ICS or the ICS/LABA combination to montelukast leads to a deterioration of asthma control [12,13]. To date, no studies have assessed a treatment step-down to different drug combinations that are delivered by different inhaler devices (dry powder and pressurised metered dose inhalers).

In terms of symptoms and pulmonary function improvement, extra-fine beclomethasone/formoterol combination therapy has demonstrated comparable efficacy to other ICS/LABA combinations containing higher nominal doses of ICS [14,15]; however, these treatments have not been compared in a step-down treatment strategy.

The aim of this study was to compare the efficacy of an extra-fine beclomethasone/formoterol combination to that of a medium dose fluticasone/salmeterol combination in maintaining lung function and asthma control following a step-down in subjects whose asthma was controlled with a high dose ICS/LABA combination.

## Methods

### Patients

This study was carried out in 67 Respiratory Clinics in Europe. Outpatients who were 18–65 years old and had been diagnosed with asthma for at least 6 months were enrolled in the study if they had been treated with 1000 mcg fluticasone propionate + 100 mcg salmeterol daily for ≥ 4 weeks before the screening visit and had features of controlled asthma, which was defined in the following manner: forced expiratory volume in one second (FEV_1_) or peak expiratory flow (PEF) values > 80% of the predicted normal values, no nocturnal symptoms or awakenings, no exacerbations, no limitations of activities, and daytime symptoms and use of rescue medication ≤ 2 days per week in the 4 weeks previous to the screening visit.

Those patients satisfying any of the following criteria were excluded: a diagnosis of chronic obstructive pulmonary disease (COPD), as defined by the GOLD guidelines [16]; current or ex-smokers (≥ 10 packs/year); a history of near fatal asthma; a symptomatic infection of the airways in the previous 8 weeks; ≥ 3 courses of oral corticosteroids or hospitalisation due to asthma in the previous 6 months, treatment with anticholinergics and antihistamines during the previous 2 weeks, or treatment with topical or intranasal corticosteroids and leukotriene antagonists during the previous 4 weeks.

The study was performed in accordance with the Good Clinical Practice guidelines recommended by the International Conference on Harmonization of Technical Requirements. The protocol was approved by the institutional review board of each centre, and written informed consent was obtained from each participant prior to the initiation of the study.

### Study design

This was a prospective, randomised, controlled, multinational, multi-centre, open, two-arm parallel group study. Asthma control was reassessed after an 8-week run-in period during which the patients continued treatment with 1000 μg fluticasone propionate/100 μg salmeterol daily. Those patients with controlled asthma in each of the last 4 weeks of the run-in were randomised to a 24-week treatment period with either 250/50 μg fluticasone/salmeterol (FP/S) Diskus DPI (Seretide®, GlaxoSmithKline, Middlesex, UK) or 100/6 μg beclomethasone/formoterol (BDP/F) pMDI (FOSTER^TM^, Chiesi Farmaceutici, Parma, Italy). The patients in the FP/S group were treated with one inhalation twice daily (daily dose 500 μg fluticasone/100 μg salmeterol). The patients in the BDP/F treatment group were treated with two inhalations twice daily (daily dose 400 μg beclomethasone/24 μg formoterol). The patients were randomised according to the pre-determined balanced-block randomisation list that was computer-generated for each centre. Concealed random allocation was done by using a fully automated functionality built into the electronic Case Report Form (e-CRF). The study group was electronically assigned to any new patient by the system and was associated to a patient number with no possibility for the study staff of changing the random allocation. Clinic visits were performed monthly for a total of 9 visits throughout the entire study. Inhaled rescue salbutamol use was permitted at any time, and oral corticosteroids were permitted only in the case of asthma exacerbation. No other anti-asthma medication was permitted at any time.

### Protocol outcome measures

The primary outcome was the change in morning peak expiratory flow (PEF) rate from baseline to the end of the treatment period (mean of weeks 23–24).

Secondary outcomes included asthma control, lung function, exacerbations, symptoms and rescue medication use.

Asthma control was assessed using the GINA composite measurements, with each week being classified as "uncontrolled", "partly controlled" or "controlled" based on the following parameters: daytime symptoms, limitation of activities, nocturnal symptoms/awakenings, use of rescue medication, lung function and exacerbations [1]. The pulmonary function tests (PFTs) were performed at each visit to the clinic in accordance with standard procedure, prior to study drug intake and at least 12 hours after the previous evening dose and 6 hours after the previous salbutamol dose. The morning dose of the study drugs was taken after the PFTs were performed at the clinic sites, under the investigator’s supervision, to assess proper inhaler technique.

The patients used a portable flow meter (Piko-1, Ferraris Louisville, CO, USA) in compliance with ATS standard 2004 to measure their morning and evening PEF values prior to the study drug intake and 6 hours from the previous salbutamol dose. The PEF values were downloaded at each visit to the study centre and directly entered in the e-CRF using dedicated software. The patients recorded asthma symptom scores (0–5) and rescue salbutamol intake twice daily on a diary card.

The investigator evaluated the occurrence of asthma exacerbations at all post-baseline visits upon reviewing the diary card with the patient, with extensive inquiry if needed. A mild exacerbation was defined as ≥ 2 consecutive days with morning PEF readings more than 20% below the baseline value, the use of > 3 additional inhalations of rescue salbutamol compared to baseline or awakening at night due to asthma [14,15]. A severe exacerbation was defined as morning PEF readings more than 30% below baseline values on ≥ 2 consecutive days or the deterioration of asthma requiring administration of oral corticosteroids [14,15]. Adverse events (AEs) were recorded throughout the study period.

### Statistics

The study was designed to evaluate the equivalence of beclomethasone/formoterol treatment and fluticasone/salmeterol treatment after step-down. The primary efficacy variable was the change in morning PEF values from the baseline values to the end of the treatment period. This variable was analysed by means of the ANCOVA model, with the treatment and the centre used as fixed factors and the baseline PEF values used as a linear covariate. Equivalence was proved if the adjusted two-sided 95% confidence interval for the mean change difference between the two treatments was entirely within the interval of −20 to +20 L/min. Estimating an alpha error set to 0.05 (two-sided), a standard deviation of 60 L/min and an expected difference between means equal to zero, a total of 191 patients were required in each group to have 90% power to satisfy the above hypothesis.

The baseline values for the variables recorded on the diary cards and the daily PEF measurements were the mean values of the last 2 weeks of the run-in period. The values that were measured in the randomisation visit (end of the run-in) were considered baseline values for the variables measured at the clinics. The last 4 weeks of the run-in period were considered to be the asthma control baseline values.

Efficacy analysis was made on intention-to-treat (ITT) population including all subjects who had been randomised to treatment and had at least one recording after randomisation. The imputation of missing primary efficacy variable data was performed using the last observation carried forward (LOCF) method for post-baseline data. Additionally, to ensure that the handling of missing data did not lead to misinterpretation, the analysis was repeated using the main ANCOVA model adopting the expectation maximisation (EM) algorithm.

P-values for the adjusted means were based on the ANCOVA model, whereas differences between treatment groups were evaluated using a chi-square test or a two-tailed Fisher’s exact test. The analysis of the time to first asthma exacerbation was performed using the Kaplan-Meier method. Safety was analysed in all of the randomised patients who received at least 1 dose of study medication. All of the statistical analyses were performed using SAS system version 9.1.3 (SAS Institute Inc., Cary, NC).

## Results

**Figure 1:**
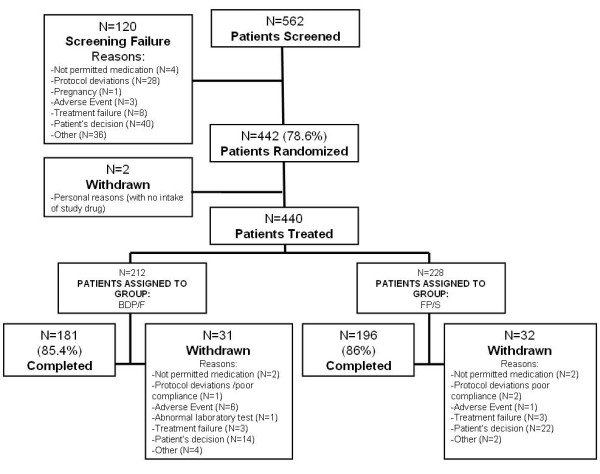
**A flow-chart representing patient flow.** BDP/F, beclomethasone dipropionate/formoterol; FP/S, fluticasone propionate/salmeterol. The reasons for screening failure and withdrawal were derived from the study termination form.

In total, 562 patients were screened between April 2007 and July 2009 (Figure1). Out of the 442 randomised patients, 440 showed evidence of study drug intake and were analysed for safety, 422 had baseline data and at least one assessment of the secondary efficacy variables after randomisation (ITT) and 378 had baseline data and at least one assessment of the primary efficacy variable after randomisation. The baseline data (Table 1) from the two groups were well matched (p > 0.05 for all comparisons). The patient compliance, as evaluated from the diary cards, was >95% in both groups during the run-in and >90% during the step-down period.

**Table 1:** Baseline characteristics of the patients examined, grouped based on randomised treatment

		**BDP/F** **400/24 μg/day (N = 206)**	**FP/S** **500/100 μg/day** ** (N = 216)**
Age (years)		44 (13)	44 (13)
Gender	Male	69 (33.5%)	77 (35.6%)
	Female	137 (66.5%)	139 (64.4%)
BMI (kg/m^2^)		27.1 (5.1)	27.0 (5.2)
Device used before screening	pMDI	12.6%	12.6%
	DPI	87.4%	87.4%
Years from asthma diagnosis		11 (10)	13 (11)
FEV1, L		2.9 (0.9)	3.0 (0.8)
FEV1% predicted		86.9 (15.1)	88.3 (14.1)
PEF, L/min		435.7 (8.3)	452.9 (8.1)
PEF % predicted		97.8 (21.4)	100.5 (20.3)
Number of days/week with symptoms^a^		0.5 (1.2)	0.3 (0.6)
Number of nights/week with symptoms^a^		0.3 (1.1)	0.1 (0.6)
Rescue medication use, puffs/week^a^		1.2 (0.4)	1.2 (0.4)
Rescue-free days/week^b^		6.6 (1.4)	6.6 (1.2)
PEF >80%, days/week^b^		5.6 (1.9)	5.5 (1.6)
Controlled asthma^b^, n (%)		203 (98.5)	212 (98.1)
Partly controlled asthma^b^, %		2 (1.0)	3 (1.4)
Uncontrolled asthma^b^, %		1 (0.5)	1 (0.5)

The values are means (SD). ^a^ the mean of the last 4 weeks of run-in period, ^b^ in each of the last 4 weeks of the run-in period. The lung function values were measured during the randomisation visit at the end of the run-in. BDP/F, beclomethasone dipropionate/formoterol; FP/S, fluticasone propionate/salmeterol; BMI, body mass index; FEV_1_, forced expiratory volume in 1 second; PEF, peak expiratory flow.

### Lung function

**Figure 2:**
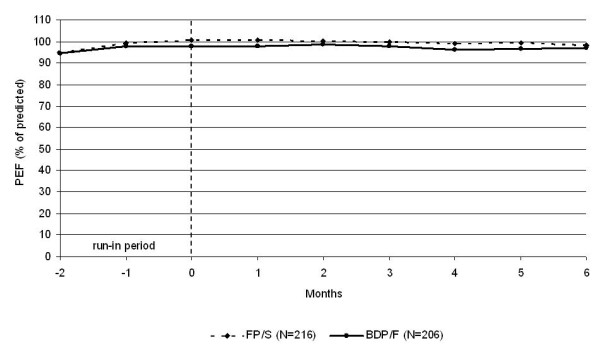
**The morning PEF % predicted values during the run-in and treatment periods (raw means).** BDP/F, beclomethasone dipropionate/formoterol; FP/S, fluticasone propionate/salmeterol.

**Figure 3:**
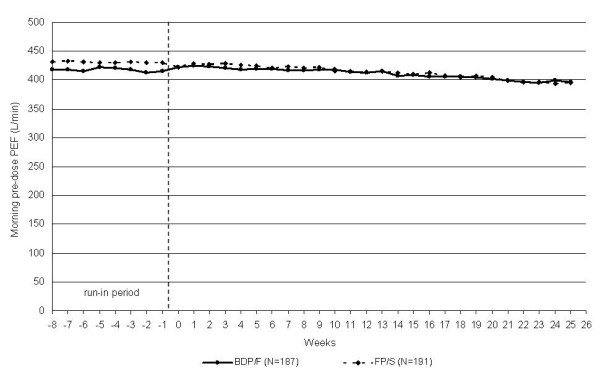
**The morning PEF absolute values during the run-in (raw means) and treatment periods (adjusted means).** BDP/F, beclomethasone dipropionate/formoterol; FP/S, fluticasone propionate/salmeterol.

The morning PEF values were equivalent in the two treatment groups [397.15 (6.47) L/min and 394.65 (6.49) L/min in the BDP/F and FP/S groups, adjusted mean (SE), respectively], exhibiting a difference of 2.49 L/min (95% CI, -13.43 to 18.42). Equivalence was also confirmed with imputation of the missing primary efficacy variable data using the expectation maximisation algorithm, which showed a difference of 1.63 L/min between the BDP/F and FP/S groups (95% CI, -14.49 to 17.76). Similarly, the per protocol analysis (including all subjects of the ITT population without major protocol deviations, n = 360) showed a 4.25 L/min difference between the treatment means (95% CI, -11.87 to 20.38). No difference between groups was detected in any lung function parameter (Table 2). The PEF values remained above 95% of the predicted values throughout the study period (Figure2). The morning PEF absolute values that were measured by patients (Figure3) at the end of the study had decreased slightly from the baseline values (from 414.4 to 397.1 L/min and from 429.7 to 394.6 L/min for BDP/F and FP/S, respectively; p < 0.05 vs. baseline for week 14 onwards in both groups). Conversely, no significant changes from baseline were detected in the PEF and FEV_1_ or the PEF and FEV_1_% of predicted values that were measured in the standardised conditions of the clinic visits (Table 3).

**Table 2:** Comparisons between groups at the end of the study

	**BDP/F** **400/24 μg/day (N = 206)**	**FP/S** **500/100 μg/day (N = 216)**	**Between group** **p value**
FEV_1_, L	2.92 (0.04)	2.92 (0.03)	0.938
FEV_1_% predicted	85.90 (0.98)	85.70 (0.98)	0.878
PEF, L/min	442.47 (4.57)	440.21 (4.55)	0.699
PEF % predicted	96.87 (1.46)	98.43 (1.32)	0.428
Daytime symptoms score^a^	1.37 (0.07)	1.32 (0.06)	0.609
Night-time symptom score^a^	1.33 (0.11)	1.46 (0.10)	0.368
symptom-free days, %	93.66 (1.13)	92.47 (1.11)	0.451
Controlled asthma^b^, n (%)	163 (90.0)	167 (85.2)	0.362
Partly controlled asthma^b^, %	11 (6.0)	18 (9.2)	
Uncontrolled asthma^b^, %	7 (4.0)	11 (5.6)	

The values are adjusted means (SE). The lung function values were measured during the last visit. ^a^ the mean of the last 4 weeks of treatment derived from diary cards, ^b^ in the last 4 weeks of the treatment period. BDP/F, beclomethasone dipropionate/formoterol; FP/S, fluticasone propionate/salmeterol; FEV_1_, forced expiratory volume in 1 second; PEF, peak expiratory flow.

**Table 3:** Changes in the lung function parameters measured at clinic visits in comparison to baseline values

	**BDP/F** **400/24 μg/day (N = 206)**	**p value**	**FP/S** **500/100 μg/day (N = 216)**	**p value**
FEV_1_, L	−0.06 (0.03)	0.089	−0.06 (0.03)	0.109
FEV_1_% predicted	−1.72 (0.98)	0.080	−1.91 (0.98)	0.051
PEF, L/min	−2.02 (4.57)	0.658	−4.28 (4.55)	0.347
PEF % predicted	−0.90 (0.96)	0.349	−1.25 (0.95)	0.190
Daytime symptoms score	−0.06 (0.07)	0.327	−0.03 (0.06)	0.661
Night-time symptom score	0.20 (0.11)	0.067	0.07 (0.10)	0.503

The values are the adjusted mean (SE) changes from baseline to the end of the study. BDP/F, beclomethasone dipropionate/formoterol; FP/S, fluticasone propionate/salmeterol; FEV_1_, forced expiratory volume in 1 second; PEF, peak expiratory flow.

### Asthma control

**Figure 4:**
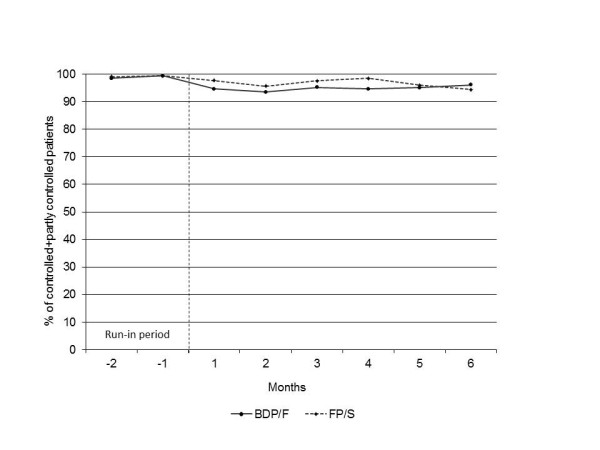
**Asthma control during the run-in period and the treatment period (raw means).** BDP/F, beclomethasone dipropionate/formoterol; FP/S, fluticasone propionate/salmeterol.

Asthma control was maintained in almost all of the patients throughout the 6-month step-down period, with the percentage of controlled plus partly controlled patients always remaining above 93% in both groups (Figure4). At the end of the study, the percentage of patients with controlled and partly controlled asthma was 96.0% in the BDP/F group and 94.4% in the FP/S group (Table 2, Figure4).

### ICS doses

A significant reduction in the mean ICS dose per week, when compared with run-in period, was found in both the FP/S group (6485.6 μg/week vs. 3182.3 μg/week during the run-in and treatment periods, respectively; p < 0.001) and the BDP/F group (6411.5 μg/week vs. 2452.0 μg/week during the run-in and treatment periods, respectively; p < 0.001). The cumulative mean ICS dose during the 6-month study period was significantly lower in the BDP/F group than the FP/S group (57.66 mg vs. 75.38 mg, respectively; p < 0.001).

### Symptoms and rescue medication use

The diurnal and nocturnal symptom scores were not different between the two groups at the end of the study, with neither group exhibiting a change from baseline (Tables 2, 3). The percent of symptom-free days (24 h) throughout the study was similar between groups (Table 2). The proportion of patients with no asthma symptoms throughout the 6-month step-down phase was 42% and 40% for daytime symptoms and 66.3% and 63.7% for night-time symptoms in the BDP/F and FP/S groups, respectively. No significant differences were detected between the groups (p = 0.69 for daytime and p = 0.61 for night-time symptoms).

**Figure 5:**
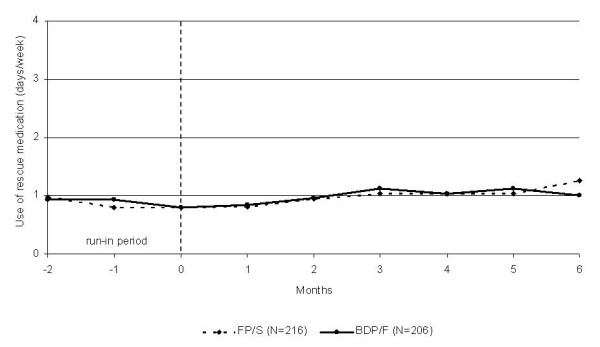
**Mean number of days/week that rescue medication was used during the run-in and treatment periods (raw means).** BDP/F, beclomethasone dipropionate/formoterol; FP/S, fluticasone propionate/salmeterol.

The use of rescue medication remained low throughout the study, and no differences were observed between the groups (Figure5). On average, rescue medication was used less than one day per week in both groups during the treatment phase. Overall, 47.8% of the patients in the BDP/F group and 44.7% of the patients in the FP/S group did not use rescue medication during the daytime of the 6-month study, and 71.7% of the patients in the BDP/F group and 70.2% of the patients in the FP/S group did not use medication during the night-time. No significant differences were observed between the groups (p = 0.56 for daytime and p = 0.75 for night-time).

### Asthma exacerbations and adverse events

Sixty-eight of the 440 patients that were evaluated for safety experienced asthma exacerbations during the 6-month treatment period. Thirty-one of these patients were in the FP/S group (14.6% of patients), and 37 were in the BDP/F group (18% of patients). Severe exacerbations occurred in 2.4% and 1.9% of patients in the FP/S and BDP/F groups, respectively. No statistically significant differences in the frequency of patients with any exacerbation (p = 0.43) or the frequency of patients with severe exacerbations (p = 1.00) were detected between the treatment groups.

The mean (SE) time to first asthma exacerbation was 154.7 (2.3) days in the FP/S group and 143.4 (2.5) days in the BDP/F group. No significant difference was observed between the groups (p = 0.36).

Adverse events (AE) were reported in 14.4% of the patients in the FP/S group and 15.5% of the patients in the BDP/F group; no differences were observed between the groups (p = 0.78). There was no difference in the proportion of patients experiencing AE that led to treatment interruption between the groups. Treatment interruption occurred in 1 patient (0.4%) in the FP/S group and 6 patients (2.8%) in the BDP/F group (p = 0.060). Serious AE occurred in 1.4% and 0.5% of patients in the FP/S and BDP/F groups, respectively. There were no differences in the proportion of patients with serious AE between the groups (p = 0.62).

## Discussion

In this study, we found that those patients who have asthma that is controlled with high dose FP/S can be stepped down to medium dose ICS/LABA combinations and still maintain asthma control. No difference in the morning PEF, asthma control, exacerbation rates and lung function measured at clinics was found between the FP/S combination and the extrafine BDP/F pMDI.

Although in clinical practice PEF is seldom used as a clinical outcome, it is easily determined and is a well-established outcome measure for monitoring asthma [1] that is consistent with previous studies, including those evaluating step-down approaches [10,11,13-15]. In our study, the PEF values were maintained at levels that were greater than 95% of the predicted values during the whole study period, which is above the 80% of predicted value required in the composite assessment of control [1]. A significant decrease in the morning PEF values of the BDP/F group (17.3 L/min) and the FP/S group (35.1 L/min) was detected 6 months after the step-down, with the latter being above the value that is considered clinically relevant [17]. This decrease in PEF values was not detected in the PFTs measured at the clinics. Such a discrepancy is consistent with the findings of the FACET study [18]. Finally, these changes were detected in both groups and are unlikely to be due to a lower compliance, as treatment compliance was greater than 90% during the step-down phase.

The current international asthma guidelines [1] recommend that treatment should be reviewed and a reduction in the dose of controller medication should be attempted once asthma control has been achieved and maintained for 3 to 6 months, with the patients being carefully monitored to ensure that control is not lost. This advice is largely based on clinical experience; few studies have examined treatment step-down options and the most favourable conditions for dose reduction. The questions that need to be addressed include whether a step-down of controller medication can be achieved without a loss of lung function and asthma control. In this study, we found that a treatment step-down that halved the ICS dose maintained asthma control and lung function in patients whose asthma was previously controlled with a high dose ICS/LABA combination. We designed the study according to the preferred option recommended by international guidelines for the step-down procedure for patients under high dose ICS/LABA consisting in reducing the ICS dose while maintaining the LABA [1].

Only a small number of studies have evaluated asthma control after treatment step-down [7,9-11,19,20], and this is the first study assessing asthma control based on the GINA guidelines [1]. We intentionally selected patients whose asthma was controlled under the highest recommended dose of the FP/S combination, which is the most prescribed therapy for obstructive lung diseases in Europe [21]. Thus, an added value of the study is that we recruited patients whose asthma was already treated and controlled with high dose ICS/LABA therapy in real life, thus identifying candidates for step-down therapy. In other words, we used a RCT to test if the high ICS doses utilised to maintain asthma control in real life conditions can be safely stepped down. Moreover, we had the advantage of examining patients over a 6-month period, a longer time frame than most of the previous studies [7,9,11,13,19,20]. Our results suggest that clinical decisions should also consider changes occurring after a prolonged period of observation (6 months).

This study is also the first to demonstrate that patients with controlled asthma can maintain control if switched from DPI to pMDI devices while stepping down treatment. The smaller particle size of the beclomethasone/formoterol combination, compared to fluticasone/salmeterol combination [22], which is formulated with larger particles, enables the ICS and LABA to reach and treat both the large and small airways, thus ensuring the treatment of inflammation and bronchoconstriction in the entire bronchial tree.

As a consequence of stepping down asthma treatment, the mean ICS dose was reduced by approximately 50% in the FP/S-receiving patients and 60% in the BDP/F-receiving patients. This reduction is of particular interest because long term treatment with high doses of ICS is associated with significant systemic side effects [1]. A recent study demonstrated that a reduction in the ICS dose could be achieved in a community setting without resulting in a worsening of airway inflammation or lung function and improved the patients’ quality of life [23].

A limitation of the present study is the open design which does not eliminate the possibility of patient and physician bias, even if the primary outcome was an objective lung function measure and data collection was independent of the investigator. Because of the modality of data collection (downloaded at each centre and entered directly in the e-CRF), the blinding of the primary outcome was not feasible. In addition, an intrinsic limitation of our study design is that we could not confirm that patients were on the minimum dose that maintained asthma control before study start. Thus the overtreatment quite commonly reported in real life conditions (8) cannot be excluded. However, patients were selected from pulmonary centres where they were regularly assessed and treated according to the current guidelines, suggesting that the dose of the original ICS/LABA prescription was appropriate. Finally, the design of our study did not allow us to evaluate whether further step-downs could have been performed in order to truly demonstrate the minimum effective dose and thus to decide whether the ICS/LABA daily dosage was appropriate.

## Conclusions

The present study demonstrated that 400/24 μg extra-fine BDP/F daily is equivalent to 500/100 μg FP/S daily in maintaining lung function and is comparable in maintaining asthma control in patients who were previously controlled with the highest recommended dose of FP/S (1000/100 μg daily). This is one of the few studies showing that asthmatic patients currently treated with combination therapy comprising high dose ICS can be equally controlled with lower exposure to ICS. Moreover, this is the first study to demonstrate that the majority of patients who are controlled with high dose ICS/LABA DPI can be stepped down to medium dose DPI or extra-fine pMDI combinations and still maintain asthma control.

## Competing interests

In the last 5 years, PLP has received grants for educational activities and institutional research from AstraZeneca, Boehringer Ingelheim, Chiesi Farmaceutici S.p.A., GlaxoSmithKline, Menarini, Merck Sharp & Dohme, Novartis, Nycomed, and Valeas. GN is employee of Chiesi Farmaceutici S.p.A. NC and DO declare no competing interests with the objective of the study. LMF was paid by BI, Chiesi Farmaceutici S.p.A., GSK, MSD, Nycomed International, Pearl Therapeutics, Sterna, Peer Voice Europe, OM Pharma Sa for consultancy work and by the offices of AZ, UCB, Novartis, Schering Plough, Sigma-Tau, Roche, Deutsches Zentrum fǖr Luft und Raumfahrt, German Aerospace Center, Mundipharma Int., Genentech Inc, Elevation Pharmaceutical, and the Ferrer Group for Advisory Board or Travel Expenses Reimbursement. LMF’s Group or Department has received Educational grants, research grants, Clinical Trial Grants, Sponsorship for Congress or Courses Organisation, from AZ, BI, Schering-Plough, Pfizer, UCB, Nycomed, Menarini Industrie Farmaceutiche, Chiesi Farmaceutici S.p.A., GSK, MSD, Roche, Novartis, Sigma-Tau, Italian Ministry for University and research, and the Italian Ministry of Health. AR has received support for research and fees for consultations, travelling and speaking from AZ, GSK, BI, Pfizer, Chiesi Farmaceutici S.p.A., Nycomed, and Philips. AP has served on Scientific Advisory Boards for, been paid lecture fees by and received research funding from AZ, Chiesi Farmaceutici S.p.A., GSK, and Mundipharma, all of which are involved in marketing ICS/LABA combination inhalers.

## Authors’ contributions

PA, NG, CN, FLM, OD, RA and PPL All of the authors contributed to the study design, data interpretation and writing of the manuscript. All of the authors read and approved the final manuscript.
